# Grain-Priming with *L*-Arginine Improves the Growth Performance of Wheat (*Triticum aestivum* L.) Plants under Drought Stress

**DOI:** 10.3390/plants11091219

**Published:** 2022-04-30

**Authors:** Hebat-Allah A. Hussein, Shifaa O. Alshammari, Sahar K. M. Kenawy, Fatma M. Elkady, Ali A. Badawy

**Affiliations:** 1 Botany and Microbiology Department, Faculty of Science (Girls Branch), Al-Azhar University, Cairo 11754, Egypt; hebahussein@azhar.edu.eg (H.-A.A.H.); saharkenawy.2052@azhar.edu.eg (S.K.M.K.); 2 Biology Department, University College of Nairiyah, University of Hafr Al-Batin, Nairiyah 31991, Saudi Arabia; 3 Biology Department, College of Science, University of Hafr Al-Batin, Hafr Al-Batin 31991, Saudi Arabia; dr.shifaa@uhb.edu.sa; 4 National Research Centre, Department of Botany, Dokki, Giza 12311, Egypt; fatma_elkady99@yahoo.com; 5 Botany and Microbiology Department, Faculty of Science, Al-Azhar University, Nasr City, Cairo 11884, Egypt

**Keywords:** stress, wheat, stimulants, photosynthetic pigments, osmolytes, phenols, flavonoids, protein pattern

## Abstract

Drought is the main limiting abiotic environmental stress worldwide. Water scarcity restricts the growth, development, and productivity of crops. Wheat (*Triticum aestivum* L.) is a fundamentally cultivated cereal crop. This study aimed to evaluate the effect of grain-priming with arginine (0.25, 0.5, and 1 mM) on growth performance and some physiological aspects of wheat plants under normal or drought-stressed conditions. Morphological growth parameters, photosynthetic pigments, soluble sugars, free amino acids, proline, total phenols, flavonoids, and proteins profiles were determined. Drought stress lowered plant growth parameters and chlorophyll *a* and *b* contents while increasing carotenoids, soluble sugars, free amino acids, proline, total phenols, and flavonoids. Soaking wheat grains with arginine (0.25, 0.5, and 1 mM) improves plant growth and mitigates the harmful effects of drought stress. The most effective treatment to alleviate the effects of drought stress on wheat plants was (1 mM) arginine, that increased root length (48.3%), leaves number (136%), shoot fresh weight (110.5%), root fresh weight (110.8%), root dry weight (107.7%), chlorophyll *a* (11.4%), chlorophyll *b* (38.7%), and carotenoids content (41.9%) compared to the corresponding control values. Arginine enhanced the synthesis of soluble sugars, proline, free amino acids, phenols, and flavonoids in wheat plants under normal or stressed conditions. Furthermore, the protein profile varies in response to drought stress and arginine pretreatments. Ultimately, pretreatment with arginine had a powerful potential to face the impacts of drought stress on wheat plants by promoting physiological and metabolic aspects.

## 1. Introduction

Different environmental stress factors disrupt plant growth, development, and productivity and induce various internal changes [1,2,3]. Drought is an abiotic stress that limits crop production across the world. The upcoming water shortage in Egypt is a real challenge facing agricultural development in general and crop production in particular. Due to this danger that threatens the future of global water, it has become necessary to use water efficiently in various agricultural and anthropogenic activities [4]. Drought induces disturbances in the photosynthetic process, development, and growth of plants and causes disorders in carbon metabolism, the carbohydrate source, leading to a partial closure of stomata with less availability of carbon dioxide [5,6]. The drought stress hampers plant growth at morphological, physiological, and molecular levels. It causes a reduction in shoot growth, area of the leaf, stomatal closure, and photosynthesis. Additionally, it induces an imbalance in nitrogen and carbon metabolism and compatible solute [7,8]. Moreover, it generates the production of reactive oxygen species, leading to membrane injury, a decline in photosynthetic rate, membrane stability, and water relations [9].

Priming of grains or seeds is one of the important physiological techniques that provides faster and synchronous germination and improves seed performance under unfavorable conditions; in addition, it is a low-cost, easy, and low-risk method [10,11]. Moreover, the use of stimulant factors to improve plant growth and productivity, even under abiotic stress, can ensure the acquisition of highly productive crops under expected climatic changes [12,13,14,15]. The application of amino acids allows plants to survive unfavorable stress conditions and stimulates their further undisturbed growth and development [2,16,17]. *L*-arginine is the functionally diverse amino acid in living cells. As it is the most versatile amino acid linked to the biosynthesis of signaling molecules, arginine plays a crucial role in stress tolerance [18]. In addition to its role as an amino acid for protein synthesis, it is an essential metabolite for various physiological, developmental, and cellular activities. Arginine is involved in the biosynthesis of proteins, proline, and polyamines and has shown its role in osmotic potential, stomatal activity, and vegetative growth [19,20]. Due to the highest nitrogen-to-carbon ratio among the 21 proteinogenic amino acids, arginine is a storage and transport form for organic nitrogen in plants [21]. Additionally, both endogenous and exogenous arginine plays a role in the plant stress response [22]. The addition of arginine induced significant increases in the growth of bean plants [23].

Wheat (*Triticum aestivum* L.) plants belong to the Graminae (Poaceae) family and are an annual, large shrub plant commonly used as food. It is an economical, valuable, and strategic cereal crop in Egypt and ranks third globally, after corn and rice, as the most productive grain [24]. It is used as a staple food grain for urban and rural societies and as a source of straw for animal feeding, so its production is directly related to the issue of food security [25]. Wheat grows and adapts to various conditions of the environment, allowing for extensive cultivation and hence storage for the long term. Generally, around 70% of wheat crops are used for human nutrition, 19% for animal feed, and the remaining 11% for industrial applications, including biofuels [15,26].

Currently, the food problem represents a challenge worldwide, particularly with limited water and land resources. Nowadays, every country should be self-sufficient in its strategic crops. To overcome the deficiency in strategic crops in Egypt, it should increase the area of the reclaimed lands and use the recent technique to increase crop productivity. Therefore, improving the qualitative and quantitative aspects of cereal crops remains a goal of many researchers [27]. Hence, the current study aims to determine the promotion role of arginine in mitigating drought stress on the growth of wheat plants via determining some morphological growth criteria, biochemical and metabolic parameters, and proteins pattern in wheat plants exposed to water deficit.

## 2. Results 

### 2.1. Growth Parameters

#### 2.1.1. Plant Height, Root Length, Tillers Number, Leaves Number, and Flag Leaf Area

It was observed in Table 1 that drought stress, arginine application, and their interactions affect the morphological growth attributes of wheat plants, represented in plant height, root length, tillers number, leaves number, and flag leaf area. Under normal conditions, application with arginine at different concentrations (0.25, 0.5, and 1 mM) caused increases in plant height, root length, tillers number, leaves number, and flag leaf area in wheat plants. Arginine at 1 mM records the most significant enhancements, reaching 14% in plant height, 59.2% in root length, 71.7% in tillers number, and 24.8% in flag leaf area, while arginine treatment at 0.5 mM caused the highest enhancement in leaves number, about 113.4% in comparison with untreated plants. Contrarily, drought stress caused a significant decrease in the plant height by 16.7% and flag leaf area by 35.5% compared to the unstressed plants, and a nonsignificant decrease in the root length, tillers, and leaves numbers. The application of arginine to drought-stressed wheat plants caused marked increases in the mentioned morphological parameters. The treatment with arginine at 0.25, 0.5, and 1 mM significantly increased the plant height of drought-stressed wheat plants by 28.13%, 29.16%, and 18.75%, respectively, compared to untreated plants. Moreover, arginine treatment at 1 mM achieved the maximum increase in the root length by 48.27% and the leaves number by 136% compared to untreated plants. However, arginine treatments caused nonsignificant increases in tillers number and flag leaf area in drought-stressed plants compared to the untreated stressed plants. 

#### 2.1.2. Fresh and Dry Weights of Shoot and Root 

Data in Table 2 showed the influence of drought stress, arginine application, and their interactions on the fresh and dry weight of shoots and roots in wheat plants. The pretreatment with arginine at different concentrations (0.25, 0.5, and 1 mM) caused marked improvements in both the fresh and dry weight of shoot and root in wheat plants grown in normal conditions. Arginine at 0.5 and 1 mM increased shoot fresh weight by 100% and 112%, respectively, while arginine at 0.25, 0.5, and 1 mM augmented the dry weight of the shoot by 52.82%, 116.75%, and 171.79%, respectively. The pretreatment with arginine at 1 mM was the most significant in the fresh and dry weight of the root, which increased by 25.1% and 34.84%, while the rest of the treatments led to insignificant increases. On the other hand, drought stress caused insignificant decreases in fresh and dry weight of the shoot, while significant in fresh and dry weight of the root per plant, represented by 59.75% and 55.22%, respectively (Table 2). Treating drought-stressed wheat plants with arginine at the tested concentrations led to marked improvements in the fresh and dry weight compared to the untreated plants. In drought-stressed wheat plants, arginine treatments at 1 mM achieved the maximum increases in the fresh weights of the shoot and root and the root dry weight by 110.53%, 110.81%, and 107.67%, respectively, compared to the untreated plants.

### 2.2. Photosynthetic Pigments

**Figure 1:**
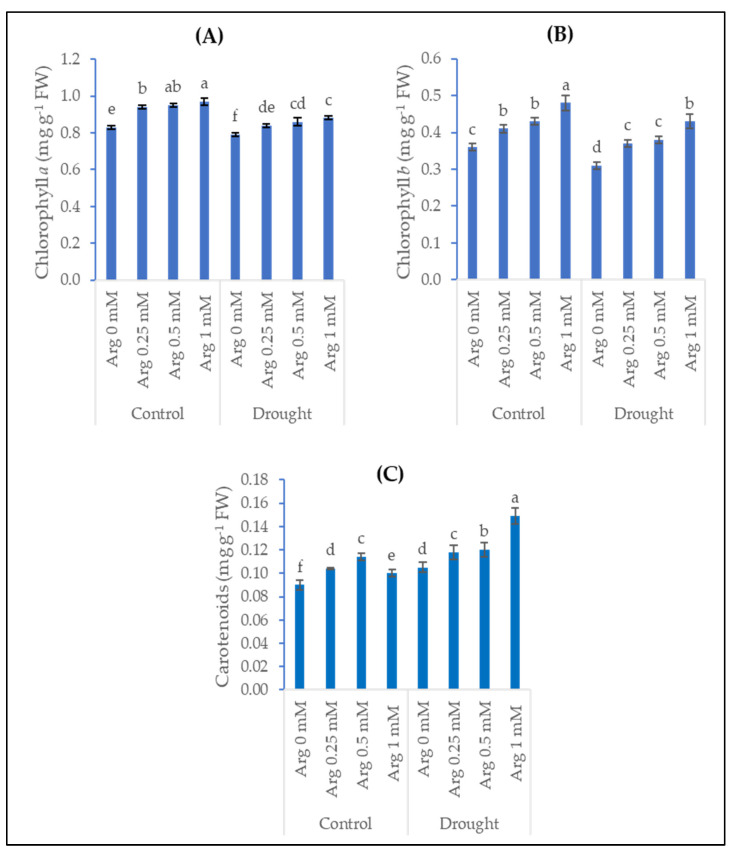
Effect of drought, arginine application, and their interactions on (**A**) chlorophyll *a*, (**B**) chlorophyll *b*, and (**C**) carotenoids contents of wheat plants. Each bar represents mean ± standard error. Means that do not share the same letter are significantly different.

The data illustrated in Figure 1 show the effect of drought stress, arginine application, and their interactions on photosynthetic pigments represented in chlorophyll *a*, chlorophyll *b*, and carotenoids in wheat plants. Different concentrations of arginine at 0.25, 0.5, and 1 mM significantly enhanced the contents of chlorophyll *a* by 13.3%, 14.5%, and 16.9%, in addition to chlorophyll *b* by 13.9%, 19.4%, and 33.3% as well as carotenoids by 15.56%, 26.67%, and 11.11%, respectively, when compared with the control (untreated plants) under normal conditions. Exposure of wheat plants to drought conditions resulted in significant inhibitions in chlorophyll *a* of about 5% and chlorophyll *b* of about 14%, while an increase in the content of carotenoids by 16.67%. Regarding drought-stressed wheat plants, the application of arginine at 0.25, 0.5, and 1 mM significantly increased chlorophyll *a* content by 6.3%, 8.9%, and 11.4%, chlorophyll *b* by 19.4%, 22.6%, and 38.7%, and carotenoids by 12.38%, 14.29%, and 41.9%, respectively, in comparison with the untreated plants. 

### 2.3. Osmo-Protectants (Soluble Sugars, Free Amino Acids, Free Proline)

**Figure 2:**
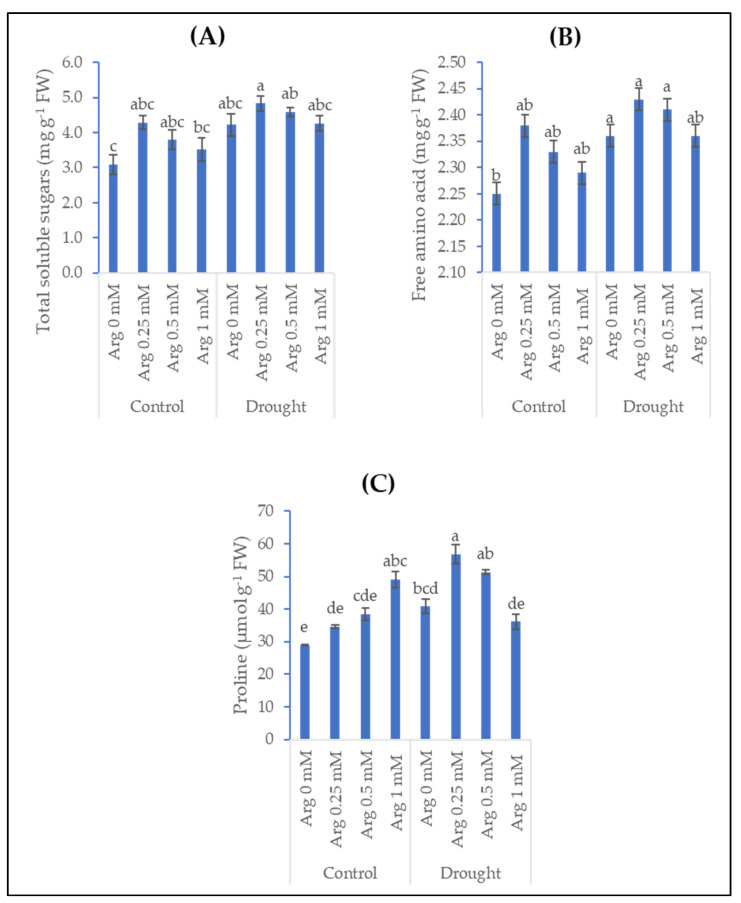
Effect of drought, arginine application, and their interactions on (**A**) soluble sugars, (**B**) free amino acids, and (**C**) proline content of wheat plants. Each bar represents mean ± standard error. Means that do not share the same letter are significantly different.

Treating wheat plants with arginine (0.25, 0.5, and 1 mM) enhanced the content of soluble sugars, free amino acids, and proline compared to untreated plants (Figure 2). The highest content of sugars and amino acids was at the lowest level of arginine (0.25 mM). A gradual increase of arginine concentration enhances proline content, and the most significant was arginine 1 mM, which recorded an enhancement in proline content of about 70%. Exposure of wheat plants to drought conditions led to an insignificant increase in total soluble sugar content of about 36.57%. However, significant increases were recorded in the amino acids and proline by 4.89% and 41.26%, respectively, compared to unstressed plants. Furthermore, the application of arginine at 0.25, 0.5, and 1 mM caused nonsignificant accumulations of soluble sugars, amino acids, and proline in drought-stressed wheat plants compared with the untreated plants, while only arginine at 0.25 mM significantly accumulated proline by 39.26%. It is worthy to note from Figure 2 that the low concentration of arginine promoted these osmo-protectants more than the high concentration in drought-stressed plants.

### 2.4. Phenols and Flavonoids

**Figure 3:**
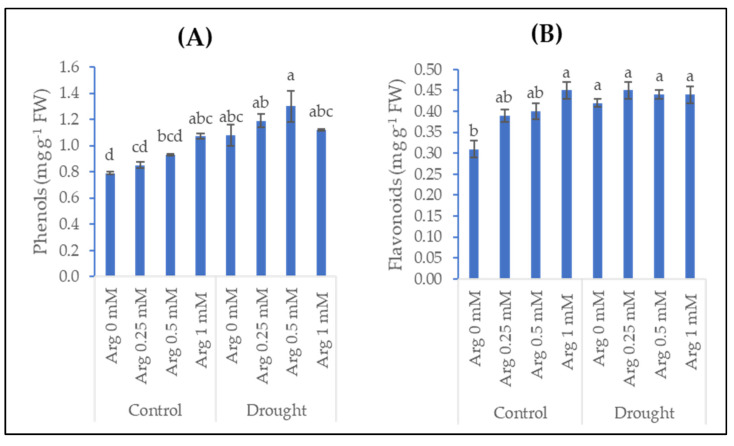
Effect of drought, arginine application, and their interactions on (**A**) phenols and (**B**) flavonoids content of wheat plants. Each bar represents mean ± standard error. Means that do not share the same letter are significantly different.

The data observed in Figure 3 clarified the impact of drought, arginine treatment, and their interactions on the contents of phenols and flavonoids in wheat plants. Utilizing arginine at different concentrations (0.25, 0.5, and 1 mM) as individual treatments gradually enhanced the contents of phenols and flavonoids in wheat plants. Arginine at 1 mM was the most effective treatment for increasing phenols and flavonoids contents. The increases reached 35.4% and 45.2%, respectively. Drought stress significantly increased the content of phenols and flavonoids by 36.7% and 35.48%, respectively, versus plants grown in normal conditions (control).

### 2.5. Protein Profile 

**Figure 4:**
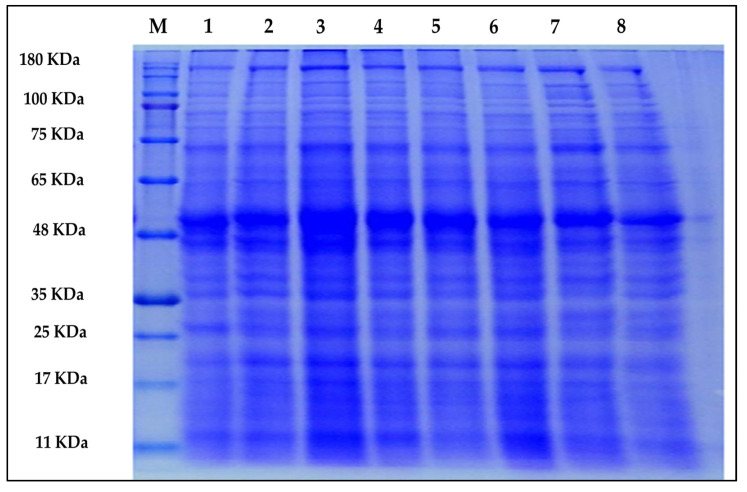
Effect of arginine pre-treatments on protein profile in flag leaves of wheat plants grown under drought stress. L1: normal control, L2: arginine 0.25 mM, L3: arginine 0.5 mM, L4: arginine 1 mM, L5: drought stress, L6: drought stress + arginine 0.25 mM, L7: drought stress + arginine 0.5 mM, L8: drought stress + arginine 1 mM.

A comparison of the protein profile of wheat plants revealed some differences in protein bands (Table 3 and Figure 4). These results showed 31 polypeptide bands with different molecular weights ranging from 12 to 180 kDa with a polymorphic ratio of 25.81%. Drought stress caused alterations in the protein profile by enhancing the synthesis or departure of different protein bands. Drought stress resulted in the synthesis of the unique polypeptide, 54 kDa in wheat plants at the vegetative stage, while the polypeptides with 14 and 15 kDa disappeared in drought-stressed plants compared to other treatments. However, arginine treatments resynthesized the missed protein bands under drought stress. Moreover, drought stress and arginine induced polymorphic polypeptides with 63, 60, 33, and 20 kDa compared to the control plants. Furthermore, the arginine treatments formed the polymorphic polypeptide, with 122 and 35 kDa in drought-stressed plants compared to the other treatments. 

Only arginine at a high concentration (1 mM) induced the appearance of the unique band at 64 KDa in drought-stressed plants. Regarding protein stabilization, there were 21 bands with different molecular weights that remained unchanged in all treatments, known as monomorphic bands. Drought stress formed a unique protein band (54 kDa) while causing the disappearance of two differentially expressed proteins (14 and 15 kDa). These results might be due to drought stress highly affecting the downregulation of three genes. On the other hand, foliar application of *L*-arginine synthesized seven different proteins and reformed two missed proteins that disappeared due to drought stress. 

## 3. Discussion

Drought stress is one of the environmental stresses responsible for limiting plant growth and productivity. In this study, drought conditions caused notable decreases in the morphological characteristics of wheat plants. The present results are in harmony with the results of some previous studies [28,29,30,31]. Water deficit reduced plant height, branches, leaves number, and leaf area in fenugreek plants [32]. A study in [33] demonstrated inhibitions in the growth criteria of the drought-stressed soybean (*Glycine max*) plants as shoot length and root length. On wheat plants, plant height, number of leaves, number of tillers, and flag leaf area were negatively affected in response to drought conditions [27]. The decrease in growth features under drought stress conditions may be attributed to tissue water loss that hinders cell division and elongation [34,35,36]. In addition, the results suggested that drought stress increased the free radical production, which subsequently alters photosynthetic pigments, protein synthesis, antioxidant status, and osmo-protectants’ accumulation. On the other hand, arginine is an essential amino acid that plays a vital role in plant growth. It caused significant increments in morphological growth characteristics of some barley cultivars [31]. Application of arginine (1 mM) on sunflower (*Helianthus annuus* L.) plants led to significant increases in lengths of the shoot and root [11]. Additionally, the application of arginine on sunflower plants promoted the shoot length and the number of leaves [37]. Moreover, pretreatment with arginine alleviated the drought effects on wheat plant growth (Table 1). Similarly, arginine treatments led to increases in plant height, number of tillers, number of leaves, and flag leaf area of drought-stressed barley plants [31]. These results may be due to the conversion of *L*-arginine into proline and nitric oxide, which is essential in plant responses to drought stress. Furthermore, the role of arginine in counteracting the adverse effects of abiotic stresses on plants may be due to the production of polyamines, which contribute to a wide range of biological processes: growth, metabolism, and abiotic stress responses [20].

Water deficit stress suppresses the fresh and dry weight of the shoot and root of wheat plants. Similarly, on maize plants, drought stress conditions reduced the fresh and dry weights of shoots and roots [38]. Furthermore, treating wheat plants with a low water supply caused suppression in the weight of shoots and roots [27]. The inhibition in the growth aspects such as fresh and dry weight may be attributed to the inability of the plant to absorb water and essential minerals under drought conditions. Thus, a disruption occurs in many vital processes, such as meristematic activity, photosynthesis, and the metabolism of carbohydrates, proteins, and lipids that are necessary for building tissues [31,39]. The pretreatment with arginine improved the fresh and dry weight of both the shoot and root in wheat plants under drought conditions (Table 1). In a previous study, exogenous application of arginine on maize plants resulted in marked increases in the fresh and dry weight of shoots and roots [22]. Furthermore, weights of the shoot and root of stressed sunflower plants significantly increased due to pre-treatment with arginine at 1 and 5 mM [11]. The stimulation effect of arginine on the fresh and dry weight of shoots and roots of water deficit-stressed plants might be due to arginine being an essential amino acid that could enhance plant growth [31].

Photosynthesis is a physico-chemical process that utilizes light energy for the biosynthesis of various organic compounds and thus plant growth. The present investigation exhibits that drought conditions reduce the contents of chlorophylls while increasing carotenoids in wheat plants. In a recent study, water deficit stress significantly reduced the chlorophylls content of peanut plants [40]. Drought stress also induced significant decreases in the content of photosynthetic pigments represented in chlorophyll *a* and chlorophyll *b* in barley plants [31]. Additionally, the application of 15% and 30% of polyethylene glycol (as a drought inducer) caused decreases in the content of chlorophyll *a*, chlorophyll *b*, and total chlorophylls in wheat plants [41]. Meanwhile, Nejadalimoradi et al. [11] reported a significant increase in carotenoids content under stress conditions. This increase might promote the antioxidant capacity because they are considered non-enzymatic antioxidants that appear under stress conditions. A study by Anjum et al. [42] reported that drought conditions damaged the photosynthetic system, reduced gas exchange, and decreased growth criteria and productivity. The decrease in the net rate of photosynthesis under drought stress could be attributed to the disorders in the biochemical processes caused by protein denaturation and lipid oxidation, which are fundamental in chloroplast and pigment structures [43]. Foliar application of amino acid arginine significantly enhanced the content of pigments (chlorophyll *a*, chlorophyll *b*, total chlorophylls, and carotenoids) in barley blades [31]. Additionally, using 0.5 mM arginine for foliar spraying on wheat plants induced increases in chlorophyll *a*, chlorophyll *b*, and total chlorophylls content [41]. Moreover, chlorophylls and carotenoid contents were promoted in sunflower leaves in response to individual foliar spray with arginine [11]. The stimulative role of arginine on chlorophyll content may be related to the structure of amino acid arginine, which acts as a nitrogen source for chlorophyll formation [18]. It is noteworthy that arginine enhanced the content of chlorophylls and carotenoids in barley plants that grow under water deficit conditions [31]. Moreover, a study by Hasanuzzaman et al. [41] indicated that the application of arginine (0.5 mM) resulted in significant increases in the content of chlorophyll *a*, chlorophyll *b*, and total chlorophylls in wheat plants when grown under the effect of 15% and 30% of polyethylene glycol (as a drought inducer). Additionally, foliar treatment with arginine at different concentrations significantly elevated leaf pigments such as chlorophylls and carotenoids in stressed plants [11,37,44]. It has been documented that the role of arginine in relieving stress and increasing the growth characteristics may be attributed to the production of polyamines, which have been implicated in a wide range of biological processes including growth, development, and abiotic stress responses [20]. 

The accumulation of compatible osmolytes is a response and mechanism from plants to reduce the oxidative damage resulting from stress as well as tolerate it. In a study on peanut plants, drought stress significantly elevated the contents of soluble sugars, proline, and free amino acids compared with their control plants [40]. Both drought stresses (15% and 30% of polyethylene glycol) significantly increased the proline content in wheat leaves compared with unstressed plants [41]. The increases in the contents of proline, soluble sugars, and free amino acids in drought-stressed wheat plants may be due to their role in the cell adaptation to drought stress conditions [12,34,45,46,47]. Such osmolytes can scavenge free radicals, inhibit cellular redox potential, adjust the osmotic pressure, stabilize membranes and proteins, and maintain the relative water content necessary for plant growth and metabolism. Additionally, the obtained results portrayed that arginine application enhanced the content of soluble sugars, free amino acids, and proline compared to untreated plants. Likewise, total soluble sugars, free proline, and total free amino acids in sunflower plants were accumulated [37]. The results suggested that an increase in carbohydrates may be due to the increases in chlorophylls and the role of arginine as a precursor for proline synthesis. Furthermore, arginine application elevated the sugars and free proline content in tomato plants grown under water deficit stress conditions [18]. Similarly, the positive effect of arginine treatment enhanced proline content in drought-stressed wheat plants, and this may be due to the fact that proline restores and regulates water and osmotic status in plants grown under stress conditions [37,41,48].

Under different environmental stresses, plants have developed various biochemical and physiological mechanisms to face or cope with these stresses. Drought stress accumulates phenols and flavonoids contents in comparison with the unstressed plants. The present results agree with [27] on wheat and [49] on pepper. They proved an accumulation of phenols content under water deficit conditions. Additionally, exposure of marigold plants to drought stress resulted in an enhancement in the content of phenols and flavonoids [50]. Furthermore, arginine treatment significantly increased the contents of phenols in sunflower plants compared with untreated plants [37]. The treatment with arginine also led to enhancements in total phenols and flavonoids contents in marigold (*Calendula officinalis* L.) plants [50]. Exogenous application of arginine is a promising technique to improve the chemical constituents and the growth of barley plants [31]. Interestingly, pre-treatment with arginine (0.25, 0.5, and 1 mM) promoted phenols and flavonoids in wheat plants grown in drought conditions compared with the untreated plants. *L*-arginine is the functionally diverse amino acid in the plant and a precursor for the biosynthesis of polyamines that play a role in stress recovery [11,44,51]. Other researchers have reported that arginine application plays a role in alleviating plant stress [20,21,52]. In a previous study, foliar treatment of arginine induced phenols and flavonoids under drought stress conditions [50]. Similarly, arginine application enhanced the content of phenolic compounds under stress conditions [37]. The promotion of phenolic compounds can alleviate the impact of drought stress on wheat plants. These findings might be due to the role of phenolic compounds as a system of antioxidant defense that can scavenge the free radicals to protect the cell from damage.

Regarding the protein profile, the appearance and disappearance of some protein bands were documented under drought conditions, and the application of arginine possibly led to the induction of some responsive genes related to drought stress tolerance. Our results predicted nine stress-responsive genes related to *L*-arginine action in adaptation to drought stress in sandy soil, and this is in agreement with previous results [53]. Generally, changes in protein expression in wheat plants exposed to drought stress and treated with *L*-arginine may have a potential role in vital physiological processes, such as polyamine synthesis, osmotic balance, membrane stability, electron transport, and signal transduction, via modulating polypeptides responsible for oxidative stress [22,54].

## 4. Materials and Methods

### 4.1. Experiment Design

A greenhouse experiment was conducted during the winter season of 2017/2018 at the Faculty of Science (Girl Branch), Al-Azhar University, Nasr City, Cairo, Egypt. Wheat grains (*Triticum aestivum* var. Sods 1) were received from the Agriculture Research Center (ARC), Giza, Egypt. Wheat grains were soaked for 12 h in the amino acid *L*-arginine (0.00, 0.25, 0.5, and 1 mM). The grains were sown on 21 November in earthenware pots No. 50 (50 cm in diameter and 50 cm in height), filled with 20 kg of sandy soil and arranged in a factorial experiment in a complete randomized design with six replicates for each treatment. After complete emergence, thinning was carried out to leave five seedlings per pot. The soil texture was sandy, field capacity 11.5%, pH 8.7, EC 0.35 dSm^−1^, Cl^−^ 1.7, HCO_3_^−^ 1.10, Na^+^ 1.2, K^+^ 0.25, Ca^++^ 1.27%, and Mg^++^ 0.58 meq L^−1^. Calcium superphosphate and potassium sulfate fertilizers were applied before sowing. Nitrogen was applied as ammonium nitrate after 30 and 60 days of sowing. At 30 days after planting, the plants were subjected to 2 irrigation regimes (100% full-field capacity as normal irrigation and 50% field capacity). Estimation of field capacity was carried out by measuring the moisture of a thin layer of soil saturated with water using a Halgard pan (weigh moist soil and dry it in an oven at 105 °C until constant, weigh the dried soil after about 24 h). Moisture at field capacity is a percentage of dry weight. Wheat plants were irrigated every 7 days. 

### 4.2. Morphological Growth Traits

At 65 days after sowing, 3 representative samples were taken from each treatment for detecting the tested growth traits: plant height (cm), root length (cm), tillers number/plant, number of leaves/plant, flag leaf area (cm^2^), as well as fresh and dry weights of shoot and root (g) per plant. 

### 4.3. Photosynthetic Pigments

According to [55], photosynthetic pigments: chlorophyll *a*, chlorophyll *b*, and carotenoids, were measured in samples of wheat fresh leaves tissues using acetone 85% and measured as detailed below. The fresh weight (0.1 g) of wheat leaves was grounded in the solvent. The homogenized samples were centrifuged at 3000 rpm, and the supernatant was up to 10 mL with acetone (85%). The absorbance was recorded at 663, 644, and 452 nm by a spectrophotometer (VEB Carl Zeiss) using acetone as a blank. The concentration of the pigment fractions (chlorophyll *a*, chlorophyll *b*, and carotenoids) was accounted for as µg/mL using the following equations:

Chlorophyll *a* = [(10.3 × A_663_) − (0.918 × A_644_)] = µg mL^−1^ (1)

Chlorophyll *b* = [(19.7 × A_644_) − (3.870 × A_663_)] = µg mL^−1^ (2)

Carotenoids = (4.2 × A_452_) − [(0.0264 × chlorophyll *a*) + (0.426 × chlorophyll *b*)] = µg mL^−1^ (3)

The concentrations of chlorophylls and carotenoids were expressed as mg g^−1^ fresh weight (FW) of plant material. The letter A refers to the absorbance.

### 4.4. Total Soluble Sugars

Total soluble sugars (TSS) were estimated in ethanol extract of fresh flag leaves of wheat plants by the anthrone technique according to [56]. TSS content was analyzed by reacting 0.1 mL of ethanol extract with 3 mL of freshly prepared anthrone (150 mg from anthrone + 100 mL from 72% H_2_SO_4_) in a boiling water bath for 10 min and reading the cooled samples at 625 nm using a spectrophotometer (VEB Carl Zeiss). Total soluble sugar was calculated using a standard curve of glucose.

### 4.5. Total Free Amino Acids

Free amino acids in fresh leaves of wheat plants were determined by the ninhydrin method according to [57], with some modifications. The ethanol extract was prepared using ethanol (80%). After centrifugation, 1 mL of extract was added to 0.5 mL of buffer 27 g sodium acetate, 20 mL distilled H_2_O, 5 mL glacial CH₃COOH, 1.5 mL NaCN (490 ppm), and completed to 75 mL with distilled water (pH = 5.4)), followed by adding 0.5 mL of ninhydrin solution (10 mg of cadmium acetate in 0.2 mL of glacial acetic acid, 0.8 mL of distilled H_2_O, and 200 mg of ninhydrin, then the reagent up to 10 mL by acetone (50%)). The previous mixture solution was placed in a water bath for 5 min until the appearance of a purple color. After cooling, 5 mL of isopropanol (50%) was added. The absorbance was measured at 570 nm using a VEB Carl Zeiss spectrophotometer. Free amino acid concentrations were calculated as mg g^−1^ FW, using a standard curve of *L*-glutamic acid. 

### 4.6. Free Proline

Proline content was determined in the tested plant samples according to [58]. Fresh leaves (0.5 g) were extracted in 10 mL of aqueous sulfosalicylic acid (3%). The extract (2 mL) was mixed with an equivalent amount of acid ninhydrin reagent and glacial acetic acid then kept for 1 h, at 100 °C. After cooling, toluene (4 mL) was added to the reaction mixture to extract the proline content. The absorbance was recorded by a spectrophotometer (VEB Carl Zeiss) at 520 nm using toluene as a blank. Free proline was determined at 458 nm from the standard curve of *L*-proline. 

### 4.7. Total Phenolics 

Total phenolic content in fresh leaves was estimated by the method described in [59,60]. One mL of the extract was added to ten drops of concentrated HCl in a boiling water bath for ten minutes and cooled. Then, 1 mL of Folin–Ciocalteau reagent and 1.5 mL of 14% sodium carbonate were mixed. The mixture was made up to 5 mL with distilled water, shaken well, and then kept in a boiling water bath for 5 minutes. The absorbance at 650 nm was noted, and the data were represented as mg g^−1^ FW using a pyrogallol standard curve. 

### 4.8. Total Flavonoids 

Total flavonoid compounds in fresh leaves of wheat plants were estimated according to [61]. A diluted ethanol extract (2 mL) was added to 0.2 mL of 5% NaNO_2_, kept for 5 min, and then reacted with 0.2 mL of 10% AlCl_3_. The absorbance was detected at 510 nm using catechin as a standard.

### 4.9. Protein Profile

The rapid freeze-dried leaf samples (0.2 g) were extracted with 1 mL of protein buffer and kept in the freezer overnight, and then vortexed for 15 s and centrifuged at 5000 rpm at 4 °C for 15 min. Then, sodium dodecyl sulfate-polyacrylamide gel electrophoresis (SDS-PAGE) was performed [62]. The molecular weight of the isolated proteins was estimated using standard molecular weight markers (standard protein markers, 11–180 kDa; Sigma, St. Louis, MO, USA). The protein bands were stained with Coomassie Brilliant Blue G-250 (Sigma, St. Louis, MO, USA) [63].

### 4.10. Statistical Analysis

Data were analyzed by using a one-way analysis of variance (ANOVA) using the Minitab^®^ 18.1 statistics data document. The results were statistically analyzed according to [64]. Tukey’s range test at a 5% level of probability was calculated to compare the means. Data were shown as mean ± standard error (*n* = 3).

## 5. Conclusions

Drought is a global problem and a limiting factor in plant growth and the performance of crops. Drought stress adversely affected the plant growth of wheat by inhibiting shoot and root lengths, tillers number, leaves number, flag leaf area, fresh and dry weights of shoot and root, chlorophyll *a*, and chlorophyll *b*. On the other hand, drought stress increased some metabolic activities as indicators of drought stress. Interestingly, soaking wheat grains in *L*-arginine is a beneficial technique to improve the growth of wheat plants under normal and drought-stressed conditions. Wheat grain-priming in arginine at 0.25, 0.5, and 1 mM, especially at a high concentration, alleviated the effects of drought via increasing the growth parameters, chlorophylls, carotenoids, osmolytes (sugars, proline, and amino acids), phenols, and flavonoids in drought-stressed wheat plants. The application of arginine induced stress-responsive genes as a mechanism for adaptation and facing drought stress. Finally, the priming technique in arginine exhibited an alleviation of drought impacts by enhancing the tolerance of wheat plants to cope with drought stress.

## Figures and Tables

**Table 1:** Effect of drought, arginine application, and their interactions on plant height (cm), root length (cm), tillers number, leaves number, and flag leaf area (cm^2^) of wheat plants. Values represent mean ± standard error. Means that do not share the same letter are significantly different.

Treatments		Plant Height (cm)	Root Length (cm)	Tillers Number	Leaves Number	Flag Leaf Area (cm^2^)
Irrigation	Arginine					
Normal	0 mM	64.0 ± 2.65 b	7.3 ± 0.33 bc	2.3 ± 0.33 ab	5.0 ± 0.58 cd	18.3 ± 0.28 ab
	0.25 mM	64.3 ± 0.33 b	8.0 ± 0.58 bc	2.0 ± 0.33 ab	8.3 ± 0.33 abc	18.6 ± 0.9 ab
	0.5 mM	67.7 ± 0.67 ab	9.0 ± 0.58 b	3.3 ± 0.33 ab	10.7 ± 1.45 a	19.3 ± 1.43 ab
	1 mM	73.0 ± 0.58 a	11.7 ± 0.33 a	4.0 ± 0.58 a	9.7 ± 0.67 a	22.7 ± 2.28 a
Drought	0 mM	53.3 ± 0.88 c	6.1 ± 0.35 c	1.7 ± 0.32 b	3.7 ± 0.33 d	11.8 ± 0.53 c
	0.25 mM	68.3 ± 1.47 ab	7.7 ± 0.33 bc	2.3 ± 0.3 ab	5.0 ± 0.58 cd	16.8 ± 0.03 bc
	0.5 mM	68.9 ± 1.45 ab	8.3 ± 0.33 b	2.3 ± 0.33 ab	6.0 ± 0.58 bcd	16.5 ± 1.32 bc
	1 mM	63.3 ± 1.42 b	9.0 ± 0.12 b	3.3 ± 0.31 ab	8.7 ± 0.33 ab	14.9 ± 0.92 bc

**Table 2:** Effect of drought, arginine application, and their interactions on shoot fresh weight (g), shoot dry weight (g), root fresh weight (g), and root dry weight (g) of wheat plants. Values represent mean ± standard error. Means that do not share the same letter are significantly different.

Treatments		Shoot Fresh Weight (g)	Shoot Dry Weight (g)	Root Fresh Weight (g)	Root Dry Weight (g)
Irrigation	Arginine				
Normal	0 mM	4.36 ± 0.3 cd	1.12 ± 0.09 d	0.92 ± 0.09 abc	0.34 ± 0.02 bcd
	0.25 mM	6.26 ± 0.37 bc	1.72 ± 0.15 c	1.08 ± 0.09 ab	0.4 ± 0.03 abc
	0.5 mM	8.72 ± 1.33 ab	2.44 ± 0.12 b	1.02 ± 0.15 abc	0.42 ± 0.02 ab
	1 mM	9.26 ± 0.49 a	3.05 ± 0.16 a	1.15 ± 0.05 a	0.45 ± 0.01 a
Drought	0 mM	2.85 ± 0.25 d	1.1 ± 0.08 d	0.37 ± 0.04 d	0.15 ± 0.02 f
	0.25 mM	4.61 ± 0.27 cd	1.16 ± 0.07 d	0.73 ± 0.04 bcd	0.22 ± 0.01 ef
	0.5 mM	5.8 ± 0.08 c	1.46 ± 0.06 cd	0.71 ± 0.03 cd	0.28 ± 0.02 de
	1 mM	6 ± 0.26 c	1.58 ± 0.04 cd	0.78 ± 0.01 bc	0.31 ± 0.01 cde

**Table 3:** Analysis of molecular weights (M.W.) of protein profile in flag leaves of wheat plants grown under drought stress and pre-treated with arginine. L1: normal control, L2: arginine 0.25 mM, L3: arginine 0.5 mM, L4: arginine 1 mM, L5: drought stress, L6: drought stress + arginine 0.25 mM, L7: drought stress + arginine 0.5 mM, L8: drought stress + arginine 1 mM.

Band No.	M.W. (KDa)	Treatments							
		L1	L2	L3	L4	L5	L6	L7	L8
1	180	+	+	+	+	+	+	+	+
2	125	+	+	+	+	+	+	+	+
3	122	-	-	-	-	-	+	+	+
4	100	+	+	+	+	+	+	+	+
5	80	+	+	+	+	+	+	+	+
6	68	+	+	+	+	+	+	+	+
7	66	+	+	+	+	+	+	+	+
8	65	+	+	+	+	+	+	+	+
9	64	-	-	-	-	-	-	-	+
10	63	-	+	+	+	+	+	+	+
11	61	+	+	+	+	+	+	+	+
12	60	-	+	+	+	+	+	+	+
13	54	-	-	-	-	+	-	-	-
14	52	+	+	+	+	+	+	+	+
15	48	+	+	+	+	+	+	+	+
16	37	+	+	+	+	+	+	+	+
17	35	-	-	-	-	-	+	+	+
18	34	+	+	+	+	+	+	+	+
19	33	-	+	+	+	+	+	+	+
20	31	+	+	+	+	+	+	+	+
21	29	+	+	+	+	+	+	+	+
22	26	+	+	+	+	+	+	+	+
23	23	+	+	+	+	+	+	+	+
24	21	+	+	+	+	+	+	+	+
25	20	-	+	+	+	+	+	+	+
26	18	+	+	+	+	+	+	+	+
27	17	+	+	+	+	+	+	+	+
28	16	+	+	+	+	+	+	+	+
29	15	+	+	+	+	-	+	+	+
30	14	+	+	+	+	-	+	+	+
31	12	+	+	+	+	+	+	+	+
Total bands		23	27	27	27	26	29	29	30

## Data Availability

The data presented in this study are available upon request from the corresponding author.
